# Attitudes of Nurses Towards Searching Online for Medical Information for Personal Health Needs: Cross-Sectional Questionnaire Study

**DOI:** 10.2196/16133

**Published:** 2020-03-16

**Authors:** Avi Zigdon, Tamar Zigdon, Daniel Sender Moran

**Affiliations:** 1 Department of Health Systems Management School of Health and Medical Sciences Ariel University Ariel Israel

**Keywords:** information retrieval, social media, evidence-based practice, nursing education, eHealth

## Abstract

**Background:**

Use of online clinical health care information has become part of the skill set required by medical teams. Nurses believe that information quality and availability affect nursing care and methods. However, nurses tend not to exploit professional medical databases for evidence-based medical information for their personal needs. This phenomenon has received little research attention.

**Objective:**

This study aimed to address the knowledge gap around nurses' attitudes towards searching online for medical information for their personal needs (ie, for themselves and their families) by (1) evaluating the level of exposure to medical information and the effect on attitudes towards the use of online search options, (2) assessing the effect of the choice of a primary means of searching for medical information on the attitudes towards the use of online search options, and (3) gauging the influence of sociodemographic data and health status on nurses’ attitudes towards searching online for medical information.

**Methods:**

Nurses employed in general departments in a general hospital (34/210, 16.2%), nursing home (42/200, 21.0%), and geriatric medical center (45/180, 25.0%) in Israel were invited to complete the eHealth Impact Questionnaire (alpha=.95). Questionnaires were distributed by nurses in charge of the general hospitalization wards. The data collection period was February to March 2018. The response rate was 40.3% (121/300).

**Results:**

Nurses tended to search for medical information for personal needs on social media (24/121, 19.8%) and TV (eg, health programs, health news; 23/121, 19.0%). Nurses who chose social media as their primary means of receiving general information had a positive attitude about using the online environment as a source for medical information compared to nurses who found information through other means (t_119_=4.44, *P*<.001). Nurses exposed to medical information via social media had a positive attitude towards the use of the internet to find medical information compared to nurses who were not exposed to social media (t_119_=3.04, *P*=.003). The attitudes of nurses towards the utility of online medical information for personal needs increased with better participant health status (*F*_2,118_=3.63, *P*=.03). However, the attitudes of participants with a chronic disease did not differ from those of healthy participants.

**Conclusions:**

Nurses in Israel are less likely to use their professional skills and knowledge to search in professional databases for evidence-based medical information for their personal needs. Instead, they prefer medical information that is easy to access and not evidence-based, such as that on social media and TV. However, these search patterns for personal use may affect their clinical role, impair quality of care, and lead to incorrect medical decisions for their patients in the health care system. Therefore, during nursing education, training for searching skills, retrieval skills, and online search techniques for evidence-based medical information is vital for evidence-based practice.

## Introduction

### Background

Searching for information using specialized, online professional databases is a required skill for medical teams in the clinical health care sector [1]. For nurses and physicians, the use of these medical databases is a legitimate part of their clinical function [2], improves patient care [3], and facilitates professional development [4] and decision-making for both patients and health care practitioners [5]. Lack of information retrieval skills and training in online search techniques [6] and the required investment in time [4,7,8] and cost [8] constitute major obstacles to searching for clinical information [7,9]. Nurses are required to possess basic knowledge and multiple skills to perform their clinical role: critical and analytical thinking, searching skills, critical reading skills, and critical evaluation of research [10]. However, nurses prefer to receive clinical information from coworkers, which may not constitute evidence-based medicine [6,11,12], with most accessing medical information in their native language [13]. On one hand, nurses acknowledge that the quality and availability of information affect nursing care and methods [9]. On the other hand, they primarily rely on Google searches [6] and mobile instant messaging applications [14], which provide non-evidence-based medical information. Bibliographic medical databases such as PubMed constitute secondary choices [6]. Nurses use online resources in their daily routines for patient care [15-17], patient training [17-19], medical monitoring [20,21], and patient health tracking [7]. In addition, nurses use virtual communities of health professionals to share professional knowledge [22]. Of course, accessing online medical information is not reserved exclusively for health professionals. Patients are active users of online medical information. With greater online accessibility comes significantly greater consumption of health information by patients [23]. Technology facilitates patient involvement and empowerment in the therapeutic process [24], promotes cooperation between patients and therapists [25], allows the medical team to elicit important feedback on patient opinions and experiences [26], and enables patient management of illness and lifestyle modification during treatment [27].

### Attitudes of Nurses Towards Searching Online for Medical Information for Personal Health Needs

Little research has focused on the use of online medical information for the personal needs of nurses, although some research has examined nurses’ use of electronic personal health records (ePHR) as health consumers [28] and social media for health needs [29-31]. In a study on factors related to the use of ePHR by nurses to manage their own health, only a third of 664 registered nurses used ePHR. This research did not find differences in demographic information, career characteristics, or healthcare experience between ePHR users and non-users. Nurses who accessed the internet for general needs used ePHR more, and electronic health (eHealth) literacy was not significantly different between ePHR users and non-users. ePHR non-users were more concerned about their privacy than ePHR users. However, a significant correlation was found between nurses who were ePHR users and nurses who had a chronic illness or underwent a drug therapy regimen [28]. Other studies found that most nurses tend not to search for health information or services when they are sick, with self-treatment very common [32] and professional roles becoming blurred with private life [1-5].

An exploratory study examining technology, internet, and social media use among nurses for personal and professional needs identified significant correlations between the likelihood of nurses recommending searching online for medical information to their patients and family members and age, level of education, and experience. An analysis between age groups found that the older group had a higher probability of recommending internet use to patients and family members. Nurses older than 30 years with formal training were less likely to recommend medical websites as an information source, while those older than 30 years without formal training were more likely to recommend internet use. Nurses with advanced nursing degrees were more likely to suggest using the internet than nurses with a bachelor's degree. Experience also had a role. Nurses with ≥31 years of reported experience had a higher chance of recommending medical websites than nurses with ≤30 years of reported experience. Only 15 nurses reported recommending their patients to “only surf” or use Google (or some other general search engine) to find medical information, while only 4 nurses suggested using .gov, .org, or .edu sources. In fact, social media use may impact the health of both individual nurses and their workplaces. Many nurses use social media for both personal and professional reasons [31], although most nurses tend not to search for health information or services when they themselves are sick, while self-treatment is very common [32].

Nurses often use social media to communicate with peers and track health-related milestones [29]. They especially favor using social media for social support and exchange of health experiences [30]. The significance of patient medical information that is available online is readily evident. This is especially the case for nurses engaged in treating patients and sometimes also for themselves and their families.

### Objectives

Very few studies have focused on nurses’ habits for personal need–based searching for medical information online. Therefore, this research examined (1) nurses’ exposure to online medical information, (2) the implications of the primary means of searching for medical information, and (3) the influence of sociodemographic data and nurses' health status on attitudes towards the use of the online environment to search for medical information.

## Methods

### Design and Setting

This research consisted of an anonymous, self-administered, cross-sectional survey based on the eHealth Impact Questionnaire (eHIQ) [33,34]. Nurses employed in the general departments of a general hospital, a nursing home, and a geriatric medical center in Israel were invited to fill out the questionnaire during the data collection period (February to March 2018). Nurses who did not work in the general department were excluded. In every medical center, 100 questionnaires were distributed by the designated head nurse in the department.

### Participants

The research participants were nurses from three general departments in various health institutions: a general hospital (34/210, 16.2%), a nursing home (42/200, 21.0%), and a geriatric medical center (45/180, 25.0%). The respondents could only fill out the questionnaire once. Every head nurse received 100 questionnaires for distribution, for a total of 300 questionnaires. Questionnaires were returned properly by 121 nurses, constituting a response rate of 40.3% (121/300).

### Statistical Analysis

Data were analyzed using SPSS Statistics version 25.0 (IBM Corp, Armonk, NY). Descriptive statistics were computed to summarize the data, with means and standard deviations calculated where applicable. The impact of exposure to medical information on the means of accessing the online environment and differences in sociodemographic characteristics were tested using one-tailed *t* tests for independent samples. Differences based ons age were tested using Chi squared tests. Differences between categories of self-reported health status were determined using one-way analysis of variance (ANOVA).

### Ethical Considerations

The research data were collected anonymously, without personal information. Answering the questionnaire involved minimal risk. Participation was voluntary. The nurses could refuse to participate in the study and stop filling out the questionnaire at any stage. The purpose of the questionnaire was explained in an introductory segment. Ethics approval was received from the Ethics Committee of Ariel University (ref AU-AZ- 20180411) before the study commenced. The head nurses in the hospitals approved the study.

### Research Tool

The eHIQ [33,34] is used to measure the effects of online health information on health consumers. The questionnaire was developed by Kelly et al [34] and verified by Kelly et al [33], with internal subscale consistency ranging from .77 to .92 (Table 1).

The questionnaire includes two parts. Part 1 consists of questions on general attitudes towards the online environment for health needs (alpha=.89). Subscale 1 (alpha=.81) measures the participant's openness to receiving online information, while Subscale 2 (alpha=.88) places emphasis on learning and receiving support from other users online. Part 2 consists of questions on the ease of use of the online environment for health needs (alpha=.93). Subscale 3 (alpha=.92) measures the level of confidence the participant has in discussing health issues with other users and identification of relevant online content. Subscale 4 measures the reliability, clarity, and level of distress felt by the participant because of online information (alpha=.62). Subscale 5 (alpha=.87) measures the ability to understand and learn from online information, along with the motivation to act accordingly (Table 1).

**Table 1:** Internal consistency of the eHealth Impact Questionnaire in this study and in the verification by Kelly et al [33].

Subscale		This study, Cronbach alpha		Verification by Kelly et al, Cronbach alpha	
**General attitudes**		0.89			
	1. Attitudes towards online health information		0.81		0.77
	2. Attitudes towards sharing health experiences online		0.88		0.89
**Ease of use**		0.93			
	3. Confidence and identification		0.92		0.92
	4. Information and presentation		0.62		0.89
	5. Understanding and motivation		0.87		0.90

We adapted and translated the English version. The questionnaire was translated into Hebrew and then re-translated into English to verify the quality of the translation and to avoid altering the meaning of the questions (alpha=.95). Face validity was tested by fellow faculty members.

For each of the scales, the sum of the answers to each item was converted from 1 to 100 according to the following formula:

((sum of scores of each item in a scale – minimum raw score) / (maximum raw score – minimum raw score)) x 100

The total score was calculated as the sum of the scores for each of the scales and the number of sub-scales:

total score = sum of subscale scores / number of subscales

In addition to the eHIQ [34], attitudes of nurses towards the reliability of online medical information and its applications were surveyed using two questions: “In general, to what extent is online health information reliable?” and “In general, to what extent is online health information useful?” These were rated on a Likert scale from 5 (to a very large degree) to 1 (not at all).

## Results

The sample consisted of 121 nurses from the three general departments in the various health institutions (nursing home, 42/121, 34.7%; geriatric medical facility, 45/121, 37.2%; and general hospital, 34/121, 28.1%). This convenience sample consisted of participants aged 24-72 years (mean 41.2 years, SD 11.4 years), with the following age distribution: 24-35 years, 45/121, 37.2%; 36-50 years, 53/121, 43.8%; ≥51 years, 23/121, 19.0% (Table 2). Of the participants, 102 nurses (102/121, 84.3%) needed medical information in the previous 2 years. Information was sought for themselves by 46 nurses (46/121, 38.0%), for first-degree relatives by 38 nurses (38/121, 31.4%), and for a second-degree relative by 30 nurses (30/121, 24.8%). Very good health was reported by 38 nurses (38/121, 31.4%), good health by 75 nurses (75/121, 62.0%), and bad or poor health by 8 nurses (8/121, 6.6%). A chronic health problem was reported by 31 nurses (31/121, 25.6%), while 89 nurses (89/121, 73.6%) reported they did not have a chronic health problem.

**Figure 1:**
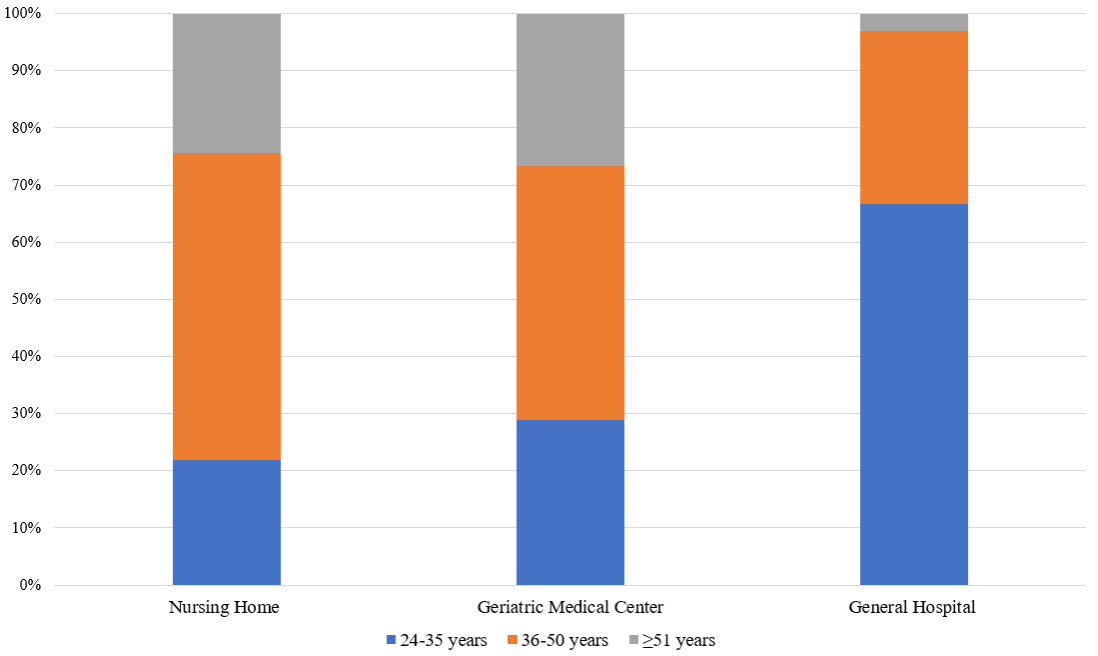
Age distribution across the three workplaces.

Figure 1 presents the distribution of age groups across the workplaces. Chi-square analysis showed a significant difference between the workplaces (χ^2^_4_=19.79, *P*<.001). Specifically, the general hospital had younger participants, while the nursing home and geriatric nursing center had more nurses aged 36-50 years.

**Table 2:** Demographic characteristics of the nurse participants (N=121).

Demographic variables		n (%)	
**Gender**			
	Female		73 (60.3)
	Male		48 (39.7)
**Age, years**			
	24-35		44 (37.0)
	36-50		52 (43.7)
	≥51		23 (19.3)
**Marital status**			
	Married/partner		74 (61.2)
	Divorced		11 (9.1)
	Widowed		4 (3.3)
	Never married		32 (26.5)
**Country of birth**			
	Israel		69 (57.0)
	Other		52 (43.0)
**Religiosity**			
	Secular		67 (55.8)
	Traditional		43 (35.8)
	Religious		10 (8.4)
**Religious affiliation**			
	Jewish		84 (69.4)
	Muslim		29 (24.0)
	Christian		5 (4.1)
	Other		3 (2.5)
**Place of residence**			
	City		92 (76.0)
	Community/locality		3 (2.5)
	Village		26 (21.5)
**Professional standing**			
	Practical nurse		9 (7.5)
	Certified nurse		52 (43.3)
	Academic nurse		59 (49.2)
**Advanced course**			
	Yes		30 (25.6)
	No		87 (74.4)

Table 3 shows the comparison between the means of searching online for medical information for personal use (self/family) and exposure to medical information online over the previous 2 years. The nurses searched for medical information for their personal needs using 2 major media platforms, namely social media (24/121, 19.8%) and TV (eg, health programs, health news; 23/121, 19.0%). Similarly, exposure to medical information on social media was reported by 19.0% (23/121) of the nurses, and exposure to medical information on TV was reported by 17.4% (21/121) of the nurses. Interestingly, 18.2% (22/121) of the participants searched for medical information by consulting a professional and not through exposure to professional medical opinion. In addition, the search for evidence-based medical information using professional journals was limited (5/121, 4.1%). Unused media included mobile phones, websites of private organizations, radio, and billboards.

**Table 3:** Comparison between the means of searching online for medical information for personal use (self/family) and exposure to medical information online over the previous 2 years (N=121). Participants could select more than one answer.

	Searching online for medical information (n=238), n (%)	Exposure to medical information online (n=283), n (%)
Social media	47 (19.7)	55 (19.4)
TV	46 (19.3)	50 (17.7)
Consulting a professional	43 (18.1)	0 (0)
News websites	28 (11.8)	34 (12)
Government websites	24 (10.1)	39 (13.8)
Service association websites	14 (5.9)	0 (0)
Non-governmental organization websites	10 (4.2)	19 (6.7)
Professional journals	10 (4.2)	0 (0)
Friends and family	9 (3.8)	0 (0)
Newspapers	7 (2.9)	13 (4.6)
Billboards	0 (0)	4 (1.4)
Radio	0 (0)	7 (2.5)
Private organization websites	0 (0)	12 (4.2)
Not exposed at all	0 (0)	13 (4.6)
Cell phone	0 (0)	37 (13.1)

No significant differences were found in the means of searching online or online exposure to medical information between the age groups (χ^2^_28_=15.00, *P*=.24)

Comparisons of the eHIQ score, subscores, and additional attitudes question scores resulted in a significant difference in the Subscale 1 (attitudes towards online health information) score, with nurses who did use social media as the primary means of accessing general information having more positive attitudes than nurses who did not use social media for that purpose (t_119_=4.44, *P*<.001). Similarly, nurses who were exposed to medical information on social media had significantly more positive attitudes according to Subscale 1 than nurses who were not exposed to medical information on social media (t_119_=3.04, *P*=.003). Nurses who accessed medical information on mobile phones had significantly more positive attitudes towards the ease of use of the online environment for health needs (t_119_=2.66, *P*=.009). Nevertheless, it was not possible to accurately determine the mode of exposure on mobile phones that allow access to social media or TV.

Nurses were also asked to select their primary means for accessing general information (one choice). In descending order, they reported using TV programs (46/121, 38.0%), social media (21/121, 17.4%), professional advice (21/121, 17.4%), news websites (11/121, 9.1%), government websites (11/121, 9.1%), non-governmental organization websites (7/121, 5.8%), and professional journals (5/121, 4.1%).

No differences were found between age groups across the media types for accessing general information (χ^2^_14_=12.66, *P*=.12). Nurses who chose social media as their primary means of accessing general information had a more positive attitude toward accessing medical information online than nurses who accessed general information by other means. Nurses exposed to medical information via social media had a more positive attitude toward using the internet to access medical information than nurses who were not exposed to social media (Table 4). Nurses who were exposed to medical information via a mobile phone had significantly more positive attitudes towards the use of the internet for medical purposes (total eHIQ score; t_117_=2.71, *P*<.001) and the ease of use of the online environment for health information (eHIQ ease of use subscore; t_116_=2.62, *P*<.001). Again, it was not possible to determine the exact mode of exposure to information via a mobile phone that allows access to social media, news websites, and professional journals. There were no other differences in the eHIQ scores based on the means to search for medical information online or exposure to medical information online.

**Table 4:** Differences in eHealth Impact Questionnaire (eHIQ) score, eHIQ subscores, and additional attitudes questions based on the use of social media as a primary means to access information online or for online exposure to medical information (N=121).

		Social media as the primary means for accessing general information				Exposure to medical information on social media			
		No (n=66), mean (SD)	Yes (n=55), mean (SD)	*t* test	*P* value	No (n=66), mean (SD)	Yes (n=55), mean (SD)	*t* test	*P* value
**eHIQ general attitudes**									
	Subscale 1. Attitudes towards online health information	46.6 (21.4)	63.5 (19.2)	t_119_=4.44	<.001	46.6 (21.4)	59.6 (22.1)	t_119_=3.04	.003
	Subscale 2. Attitudes towards sharing health experiences online	55.0 (24.0)	64.5 (22.6)	t_117_=2.15	.03	55.0 (24.0)	61.7 (24.0)	t_117_=1.22	.22
	Total score	50.4 (19.8)	64.0 (18.6)	t_117_=3.74	<.001	50.4 (19.8)	60.4 (20.4)	t_117_=2.31	.02
**eHIQ ease of use**									
	Subscale 3. Confidence and identification	47.9 (18.2)	65.4 (16.6)	t_117_=5.31	<.001	47.9 (18.2)	63.7 (16.7)	t_117_=4.88	<.001
	Subscale 4. Information and presentation	45.9 (18.9)	63.7 (18.9)	t_116_=4.99	<.001	45.9 (18.9)	61.5 (19.2)	t_116_=4.45	<.001
	Subscale 5. Understanding and motivation	49.0 (18.9)	66.8 (18.2)	t_117_=5.10	<.001	49.0 (18.9)	64.7 (18.9)	t_117_=4.43	<.001
	Total score	47.6 (17.0)	65.3 (17.0)	t_116_=5.53	<.001	47.6 (17.0)	63.3 (17.2)	t_116_=4.97	<.001
eHIQ total score		48.6 (16.8)	64.8 (16.9)	t_115_=5.07	<.001	48.6 (16.8)	62.2 (17.6)	t_115_=4.10	<.001
**Additional attitudes questions**									
	Reliability of internet information	3.2 (0.9)	3.8 (0.7)	t_119_=3.68	<.001	3.2 (0.9)	3.7 (0.8)	t_119_=3.68	<.001
	Usefulness of internet information	3.3 (0.8)	4.0 (0.8)	t_119_=4.55	<.001	3.3 (0.8)	3.9 (0.8)	t_119_=4.55	<.001

Regarding the effect of sociodemographic characteristics on nurses' attitudes, attitudes towards accessing information online were significantly different by place of birth and living area.

Nurses born in Israel had a significantly lower mean eHIQ general attitude score (mean 51.115, SD 20.636) than nurses not born in Israel (mean 62.194, SD 18.317; t_117_=3.028, *P*=.003). Nurses born in Israel also had a significantly lower Subscale 1 (attitudes towards online health information) scores (mean 49.114, SD 22.030) than nurses not born in Israel (mean 58.440, SD 21.205; t_119_=2.342, *P*=.02). In addition, nurses born in Israel had a significantly lower mean eHIQ Subscale 2 (attitudes towards sharing health experiences online) score (mean 53.116, SD 24.706) than nurses who were not born in Israel (mean 66.500, SD 20.335; t_117_=3.136, *P*=.002). There were no statistically significant differences in the eHIQ ease of use score, attitude toward information reliability, or attitude toward information usefulness based on country of birth.

Based on place of residence, nurses living in the center of Israel had a significantly higher mean eHIQ general attitude score (mean 58.422, SD 19.589) than nurses living in the peripheral areas of Israel (mean 48.244, SD 20.973; t_117_=2.442, *P*=.02). Nurses living in the center of Israel also had a significantly higher Subscale 1 (attitudes towards online health information) score (mean 55.741, SD 21.836) than nurses living in the peripheral areas of Israel (mean 45.520, SD 21.345; t_119_=2.260, *P*=.03). In addition, nurses living in the center of Israel had a significantly higher mean eHIQ Subscale 2 (attitudes towards sharing health experiences online) score (mean 61.477, SD 21.674) than nurses living in the peripheral areas of Israel (mean 50.968, SD 28.030; t_117_=2.144, *P*=.03). No significance differences were found based on place of residence in the attitudes towards security and identification (Subscale 3), reliability of online health information, and usefulness of online health information. There were also no differences in nurses’ attitudes towards searching online for personal need–based medical information based on the remaining sociodemographic characteristics.

The attitude towards the usefulness of online health information was more positive with increasing self-reported health (not so good, good, and very good; Table 5). However, the attitude was not different between participants who had a chronic disease and those who did not have a chronic disease.

There was a significant correlation between the attitudes towards the reliability of online information and attitudes towards the usefulness of online information (r=.758, *P*<.001).

**Table 5:** Attitudes towards the usefulness of online information according to self-reported level of health (N=121).

	Not so good (n=8), mean (SD)	Good (n=75), mean (SD)	Very good (n=38), mean (SD)	F statistic	*P* value
Usefulness of online health information	3.3 (0.7)	3.4 (0.8)	3.8 (1.0)	*F*_2,118_=3.63	.03

## Discussion

### Principal Findings

Evidence-based medical information is a main resource for medical teams in health care systems. Only a few studies have examined the attitude of nurses towards searching online for personal need–based medical information. This research sheds light on their attitudes, showing that nurses mainly use social media (24/121, 19.8%) and TV (eg, health programs, health news; 23/121, 19.0%) for this purpose. The general attitude of participants who chose/were exposed to social media as the main source of medical information was significantly more positive in comparison to those who did not choose/were not exposed to social media as a source of medical information. Attitudes towards the ease of use of the online environment for health needs were also significantly greater for nurses who accessed health information via mobile phones than for those who did not use mobile phones to access information (t_119_=2.66, *P*=.009). Nevertheless, it is not possible to accurately determine the method of accessing information on a mobile phone that allows access to social media and TV.

Some nurses search for medical information by consulting a professional (22/121, 18.2%). However, they express only marginal interest in using professional research sources (5/121, 4.1%) in their personal need–based medical information searching. This is consistent with Wolf et al [31], who found that only 4 nurses recommended to their patients and family members the use of .gov, .org, or .edu sources.

Research would be expected to find that nurses, as professional health care practitioners, look for personal need–based medical information using medical databases such as PubMed. This would align with the most prudent path of decision-making for their health, but the reality is quite different. Other research data on searching for professional health care information by nurses for patient treatment [15-17], patient training [17-19], medical monitoring [20,21], and tracking patient health [7] indicate that nurses prefer to access clinical information from co-workers [6,11,12], information which is not necessarily evidence-based [6].

The results of this study also show that using mobile phones for nurses' personal need–based medical information was linked to a more positive attitude toward the ease of use of the online environment for health information. However, a mobile phone represents a means of accessing the online environment and does not constitute an information source. In other studies, mobile instant messaging apps [14] were a way for nurses to search for information, track milestones related to their health [29], and, especially, for social support and exchange of health experiences [30]. In this study, nurses' attitudes towards using the online environment to obtain medical information for their personal needs were significantly different based on place of birth and their residence; there were no differences based on the other sociodemographic characteristics. Self-reported health status and chronic disease did not affect nurses' preferences for the source of health information, which differs from the findings of Wolf et al [31] who found that with older age, nurses with no formal training and experienced nurses alike tended to recommend the internet for medical purposes for their patients. This finding is supported by the assumption that a patient with a disease receives orderly medical care, whereas an individual experiencing clear symptoms but has not yet undergone a medical examination will actively search for online medical information. This assumption should be examined in future studies.

### Limitations and Future Research

This study contains some methodological limitations. First, the nurses filled out the questionnaires in the workplace where they provided patient treatment. This could subjectively affect the responses as the questionnaire deals with individual attitudes in the use of online medical information for personal needs. At the same time, health status is an issue that has been reviewed by nurses’ personnel reports. The medical conditions of the participants were not verified, and it is unclear whether the severity of a medical condition affected the reported attitudes. In addition, it is difficult to determine the exact mode of participant exposure to information when using a mobile phone, as it enables access to social media, news websites, and professional research. To provide further focus in the results, targeted research is needed, and health reporting based on disease codes from medical files and subjective reports is required.

### Conclusion

Nurses in Israel tend not to use their professional skills and knowledge to search for evidence-based medical information using a professional database such as PubMed when looking for medical information for themselves and their families. They prefer non-evidence-based medical information that is easy to access such as that found on social media and TV. These search patterns for information for personal use may affect their clinical role, impair the quality of care, and lead to incorrect medical decisions for their patients in the health care system. Moreover, these patterns might hinder their professional development and establish consumption patterns of erroneous medical information. Therefore, during nursing education, training for searching, retrieval skills, and training in online search techniques for evidence-based medical information is vital for evidence-based practice. A change to information-seeking behaviors that focus on evidence-based information can be tested in a government-issued standardized exam directly following formal training, and 6-monthly seminars on advanced searching skills for medical information can be offered.

## Abbreviations

eHealth: electronic health.
eHIQ: eHealth Impact Questionnaire.
ePHR: electronic personal health records.
